# Comparative Outcomes of Robotic Radical Prostatectomy in Patients with Locally Advanced Prostate Cancer

**DOI:** 10.3390/medicina58121820

**Published:** 2022-12-10

**Authors:** Po-I Li, Szu-Ju Chen, Yung-Hsiang Chen, Wen-Chi Chen, Chi-Ping Huang

**Affiliations:** 1 Department of Urology, China Medical University Hospital, Taichung 40447, Taiwan; 2 Division of Urology, Department of Surgery, Taichung Veterans General Hospital, Taichung 40705, Taiwan; 3 Graduate Institute of Integrated Medicine, College of Chinese Medicine, China Medical University, Taichung 40402, Taiwan; 4 Department of Psychology, College of Medical and Health Science, Asia University, Taichung 41354, Taiwan; 5 School of Medicine, College of Medicine, China Medical University, Taichung 40402, Taiwan

**Keywords:** locally advanced prostate cancer, robotic-assisted radical prostatectomy, outcome

## Abstract

The effectiveness of radical prostatectomy alone for locally advanced prostate cancer is controversial owing to an increased complication rate and treatment-related morbidity. With technical advances and refinements in surgical techniques, robotic-assisted radical prostatectomy (RARP) has improved the outcomes of patients with locally advanced prostate cancer. RARP therefore plays a role in the treatment of locally advanced prostate cancer. In this study, we enrolled a total of 76 patients with pathologic stage pT3a, pT3b, pT4, or pN1. All patients were followed from surgery to June 2022, and their characteristics, perioperative outcomes, complications, adjuvant therapies and outcomes were analyzed. The median age of the patients was 69 years, and the initial PSA level was 20.5 (IQR 10.8–31.6) ng/mL. The median operative time was 205 (IQR 182–241) minutes. Sixty-six patients (86.8%) regained continence within 1 year, and the continence rate within 3 years of follow-up was 90.8% (69 patients). The overall survival rate was 100%. Twenty-two patients had BCR, of whom 13 received salvage androgen deprivation therapy (ADT), 2 received salvage external beam radiation therapy (EBRT) alone, and 7 received combined ADT and EBRT. No patient had disease progression to castration-resistant prostate cancer during a median 36 months of follow-up after salvage therapy. Our results suggest that RARP can also decrease tumor burden and allow for accurate and precise pathological staging with the need for subsequent treatment. Therefore, we recommend that RARP represents a well-standardized, safe, and oncologically effective option for patients with locally advanced prostate cancer.

## 1. Introduction

Prostate cancer is the most common male genitourinary malignancy, especially in Western countries [1,2]. There is also a trend of an increasing incidence of prostate cancer in Taiwan [3], where the incidence increased from 35.47 in 2006 to 72.81 in 2016 per 100,000 men. In addition, prostate cancer was the eighth most common cancer in men in 1998, and the fifth most common cancer in men from 2013 until now [4]. In contrast, there has been a decline or stabilization in the incidence and mortality in many countries, especially in high-income countries, due to universal prostate-specific antigen (PSA) testing [5]. After a diagnosis of prostate cancer, treatment options are based on risk stratification [6], including PSA value, staging, Gleason score, and cancer nomogram [7,8,9,10,11].

Traditionally, radical prostatectomy (RP) alone has been considered a good treatment option for locally confined prostate cancer [6]. Compared with watchful waiting, Bill-Axelson et al. reported that surgical treatment had good long-term outcomes with a reduction in mortality rate over a 23-year study period [12]. However, the effectiveness of RP alone for locally advanced prostate cancer is controversial owing to the increased complication rate and treatment-related morbidity [13]. In 2003, a European consensus stated that optimal treatment of locally advanced prostate cancer should be individually tailored according to PSA, clinical staging, and Gleason score [14]. For the treatment of locally advanced prostate cancer, a systematic review of 90 studies conducted in 2020 found good evidence to recommend RP [15]. Furthermore, with technical advances and refinements in surgical techniques, robotic-assisted radical prostatectomy (RARP) has improved the outcomes for patients with locally advanced prostate cancer [16,17].

Despite the uncertain effectiveness of RP alone for locally advanced prostate cancer, it can decrease tumor burden and allow for the accurate and precise pathological staging with the need for subsequent treatment. Mazzone et al. reported technical refinement of the procedure (super-extended RARP) by dissecting the Denonvilliers’ fascia and perirectal fat and leaving it on the posterior surface of the seminal vesicles [18]. They demonstrated that this technique was feasible for patients with locally advanced prostate cancer, with good outcomes, good continence recovery rate, and a delay in the use of additional treatments and biochemical control. Based on the previous work, we share our experience of RARP for patients with locally advanced (≥pT3 and any pTN1M0) prostate cancer.

## 2. Materials and Methods

### 2.1. Patients and Study Design

This is a single-center, single-surgeon retrospective review of patients with locally advanced prostate cancer who underwent RARP from January 2014 to December 2019 at China Medical University Hospital. All patients were diagnosed using transrectal sonographic-guided biopsy. Preoperative imaging included abdomen-computed tomography, multiparametric magnetic resonance imaging (mpMRI) with analysis of PI-RAD score [19], and bone scan. Patients with pathologic stage pT3a, pT3b, pT4, or pN1 were enrolled. We excluded patients with boney metastasis from this study.

Under general anesthesia, the patients were placed in the steep Trendelenburg position (with their head down at an angle of 25°). RARP was performed using a 6-port transperitoneal approach (4-arm configuration and 2 additional assistant ports) in all patients with a Da Vinci Si Surgical System (Intuitive Surgical, Sunnyvale, CA, USA). We performed lymph node dissection first, with extended pelvic lymph node dissection according to the procedures described by Gandaglia et al. [16], which included the intrapelvic area (obturator, internal and external iliac) and the common iliac area up to bilateral ureteric crossings.

For wide resection of the surrounding prostate tissue, Denonvilliers’ fascia was incised during isolation and dissection of the seminal vesicles. Perirectal fatty tissue was entered and dissected between the rectum and the posterior aspect of Denonvilliers’ fascia. Denonvilliers’ fascia was then completely dissected and left on the posterior surface of the seminal vesicles. Dissection of the lateral extrafascia to the levator ani fascia was then performed, followed by bladder detachment, endo-pelvic fascia incision, bladder neck incision, ligation of the dorsal venous complex, apical dissection, posterior reconstruction, and urethra-vesical anastomosis, according to the recommendations of best practice for RARP reported by Montorsi et al. [20].

The decision of whether to spare neurovascular bundles was made according to the findings of mpMRI, in which a side was not affected by tumor on either the unilateral or bilateral side. Otherwise, all neurovascular bundles were dissected and excised with the capsule.

### 2.2. Statistical Assay

All patients were followed from surgery to June 2022, and their characteristics, perioperative outcomes, complications, adjuvant therapies and outcomes were analyzed. The biochemical recurrence (BCR) rate was defined as an increase in PSA > 0.2 ng/dL after RARP [21]. Survival and BCR were analyzed using Kaplan–Meier survival curves.

The patients’ demographic data including age, initial prostate-specific antigen (iPSA) level, body mass index (BMI), days of hospital stay, Gleason score, D’Amico risk classification, and comorbidities were collected and analyzed. Perioperative outcomes including operative time, blood loss, rate of nerve sparing, rate and number of lymph node dissection, and complications were also analyzed. We divided the patient’s tumor stage into IIIb, IIIc, and IV to calculate the positive margin-free rate. The continence-free rate was evaluated at 1 year and 3 years post-operation. Adjuvant hormone therapy and radiotherapy were calculated. Kaplan–Meier survival analysis for the BCR was performed.

## 3. Results

A total of 153 patients with prostate cancer underwent RARP and bilateral pelvic lymph node dissection (BPLND) during the study period, of whom 76 had locally advanced prostate cancer. The median age of the patients was 69 years (interquartile range [IQR] 64–75 years), and the initial PSA level was 20.5 ng/mL (IQR 10.8–31.6 ng/mL). All patients were followed until June 2022, and the median follow-up duration was 36 months (IQR 29.8–50.3 months). All patients included in this study were of pathology stage T3, T4, or N1. Table 1 shows the demographic data of the included patients, of whom 55 were in the D’Amico high-risk group and 12 were in the very high-risk group. With regards to comorbidities, 34 (45%) had diabetes mellitus, 16 (21%) had hypertension, 7 (9%) had cardiovascular disease, and 3 (4%) had cerebrovascular accidents (Table 1).

Table 2 presents the perioperative outcomes of RARP. The median operative time was 205 min (IQR 183–241 min). The median blood loss was 50 mL (IQR 50–70 mL), and no blood transfusions were required during surgery. The median length of hospital stay was 7 days (IQR 7–9 days). Overall, two patients had Clavien–Dindo grade I complications and one patient had a Clavien–Dindo grade IV complication; this patient was transferred to the intensive care unit due to choking caused by aspiration pneumonia. Sixty-six patients (86.8%) regained continence within 1 year, and the continence rate at 3 years of follow-up was 90.8%.

**Figure 1:**
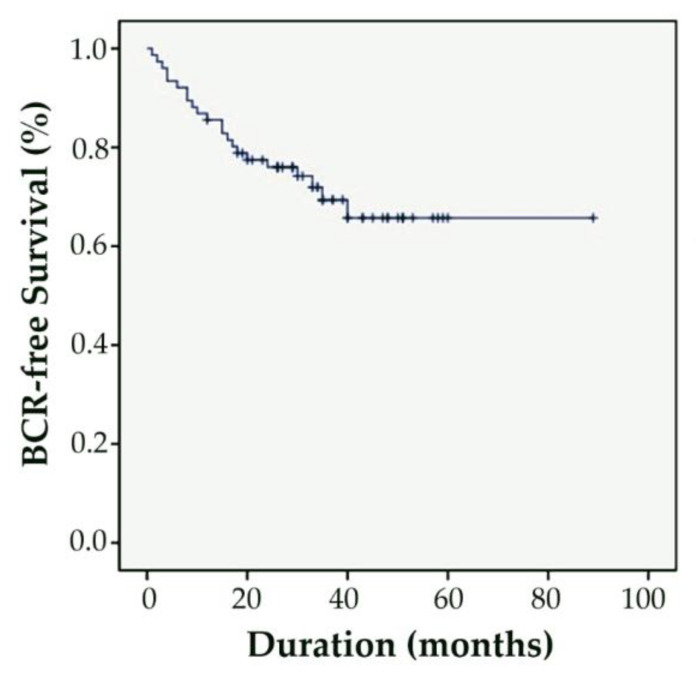
Kaplan-Meier analyses assessing time to BCR in patients with locally advanced prostate cancer treated with RARP and BPLND.

During a median 36 months of follow-up, the BCR-free rate and overall survival rate were 71.1% and 100.0%, respectively. None of the patients died of cancer-specific causes. Twenty-two patients had BCR, of whom thirteen received salvage androgen deprivation therapy (ADT), two received salvage external beam radiation therapy (EBRT) alone, and seven received combined ADT and EBRT. No patient had disease progression to castration-resistant prostate cancer during a median 31 months of follow-up after salvage therapy (Figure 1).

## 4. Discussion

Our results are comparable to previous studies of RARP for locally advanced prostate cancer with respect to blood loss, low complication rate, good continence control, and BCR rate. In a review by Saika et al. of several reports regarding the operative time for RARP in patients with locally advanced prostate cancer, the time ranged from 200 to 271 min [22]. This is consistent with our result of an average of 205 min. However, blood loss in our series was less than in their report, and no blood transfusions were needed. Our patients were older and had a higher PSA level and pathologic stage but a relatively lower complication rate, with only three patients having Clavien–Dindo classification I complications. For the perioperative outcomes, we had excellent results with regards to the number of nodes removed, 3-year BCR-free rate, overall survival rate, and continence rate. In addition, the rate of adjuvant radiotherapy after BCR was lower in our study. Furthermore, only one patient died during the follow-up period, but this was not caused by cancer. Moreover, among 22 patients with biochemical failure who received salvage therapy, there was no disease progression to castration-resistant prostate cancer during a median 31 months of follow-up. Therefore, our results support RARP as a treatment option for patients with locally advanced prostate cancer.

There is debate regarding the use of radical prostatectomy as a first-line treatment for locally advanced prostate cancer. According to the National Comprehensive Cancer Network (NCCN) guidelines, locally advanced prostate cancers are defined as those with at least one of the following factors: pT3b-T4 disease, pathology primary Gleason pattern grade 5, or more than four positive cores of Gleason score 8–10 [23]. The primary treatment recommendations include five options: ADT alone, ADT with EBRT, EBRT with brachytherapy with and without ADT, EBRT with ADT, and docetaxel (or RP and BPLND in select patients). RP is an appropriate option for patients who have a life expectancy greater than 10 years without serious comorbid conditions. An increasing number of studies have demonstrated that RP alone is beneficial and provides survival outcomes for patients with locally advanced prostate cancer [24,25]. Tilki et al. conducted a comparative analysis between patients with different treatment options, and of 372 men treated with RP alone, 71 (19%) had died at a median follow-up of 4.78 years [26]. Although our follow-up period was only 3 years, our results also support that RARP may be a suitable first-line treatment for locally advanced prostate cancer.

Gagnon et al. compared open prostatectomy (OP) and RARP with 200 patients in each group in 2014, and found that the OP group had a high proportion of locally advanced prostate cancer and shorter operative time than the RARP group [27,28]. There were comparable results with respect to transfusion rate, length of hospital stay, positive margin rate, incontinence rate, and 1-year biochemical-free status. The complication rate was lower in the OP group than in the RARP group (8.5% vs. 20.0%). However, with technical improvements in RARP, and the surgeon’s experience, Simsir et al. reported initial favorable results of RARP in 204 patients compared with 755 patients who underwent OP [29]. The results of transfusion rate, length of hospital stay, urine leak rate, and complication rate in the RARP group were better than those in the OP group. However, the operative time was shorter in the OP group than in the RARP group (117 vs. 188 min). The operative time in our study was longer than in these reports, possibly due to the different stages of prostate cancer and the extended lymph node dissection. However, our results indicating low complication rate, high continence rate, and 1-year BCR rate were comparable.

Regarding return to continence, we had a high success rate of 86% within 1 year, and 90% within 3 years of follow-up. Fukui et al. reported that a pre-operative evaluation of the membranous urethral length (MUL) and a pubic symphysis prostate apex index (PAL) using mpMRI could be used to predict continence rate, with a long MUL and a short PAL being associated with a high continence rate 3 months after surgery (60% vs. 31%) [30]. Although we performed mpMRI before surgery without measuring the MUL, we analyzed the continence rate at 1 year and 3 years of follow-up instead. However, their patients had locally confined prostate cancer, and follow-up data at 1 year were not reported. We may use this prediction method in the future to analyze the continence rate earlier, within 3 months.

With regards to pre-operative imaging using mpMRI, the use of radiological diagnostic tools is important. For example, a Prostate Imaging Reporting and Data System (PI-RAD) score over three has a high diagnostic value for prostate cancer. However, whether to perform a biopsy in those with a score of three is unclear. Recently, Gravina et al. reported the use of machine learning to aid in the diagnosis [31]. The parameters included BMI, serum PSA, location of PI-RAD score three, prostate volume, PSA density, and results of histopathology, etc. Four machine learning models were developed: a classification tree, random forest, support vector machine, and feedforward neural network. All models showed good validity in the prediction of whether to perform a biopsy. In addition, a meta-analysis of 12 studies regarding machine learning for the identification of clinically significant prostate cancer found promising results, with an area under the curve of 0.86 [32]. This high accuracy may help to improve the diagnostic rate of prostate cancer, and further investigations are warranted.

Most complications of RARP are mild. Fuller et al. reported a 22.9% (70/305 patients) complication rate of RARP, of whom 67.1% (47 patients) had Clavien I-II complications and only required conservative treatment [33]. The complication rate was also low in our study (3.9%), which is comparable with the intraoperative complication rate reported by Mazzone et al. (3.4%) [18]. In addition, they reported a 2-year BCR rate of 55.0%, compared to 71.1% in this present study. Therefore, RARP appears to be safe and beneficial for patients with locally advanced prostate cancer.

Demoralization in patients with locally advanced prostate cancer may have an impact on their health, such as resilience after surgery. Mental health issues after a diagnosis of prostate cancer, such as depression and masculine self-esteem, can lead to demoralization. The psychophysiological aspects of patients with locally advanced prostate cancer undergoing RP and/or radiotherapy were evaluated by Scandurra et al. [34]. They studied the mental health of 197 patients with locally advanced prostate cancer using questionnaires including the abovementioned psychological parameters, and found a linear relationship between demoralization and depression, i.e., higher depression scores were associated with higher demoralization scores. High demoralization was also associated with a lower level of masculine self-esteem. Resilience significantly protects from the effects of low masculine self-esteem in patients with prostate cancer. Therefore, it is important to pay attention to the patient’s mental health, such as masculine self-esteem, depression, and demoralization when treating patients with prostate cancer. Good operative outcomes may also increase a patient’s resilience.

Blood loss is an important concern during RARP. Di Bello et al. studied red blood cell count, hemoglobin (Hb), and hematocrit in 363 patients with prostate cancer who underwent RAR [35]. In the peri-operative period, there was a short-term significant decline in Hb of approximately 3 points between the pre-operative period and 2 days after surgery. This decline was limited and stabilized 3 days after surgery. This post-operative change in red blood cell count should remind us to pay attention to the quality of post-operative care. In our series, the average blood loss was 66.84 ± 56.20 mL, which may reflect this trend.

There is a shift from open prostatectomy to RARP for the surgical treatment of locally advanced prostate cancer. Despite the aforementioned benefits of RARP compared to OP, including short hospital stay, less blood loss, low complication rate and good continence rate, there are still several disadvantages to RARP, including a steep learning curve, high cost, and need for specialized equipment.

The limitations of this study include the limited number of patients, short follow-up period, and experience from only a single medical center and a single surgeon. However, we achieved a high operative success rate, low complication rate, and good tumor outcomes in a short-term follow-up period. Further studies should investigate the cancer survival rate over a longer follow-up period in more patients.

## 5. Conclusions

RARP represents a well-standardized, safe, and oncologically effective option for patients with locally advanced prostate cancer. Although the number of patients was limited, and the experience was only from a single surgeon, the results were promising. We recommend RARP as a treatment option for select patients with locally advanced prostate cancer. Further studies with more patients are warranted to verify our results.

## Figures and Tables

**Table 1:** Demographic characteristics of patients with locally advanced prostate cancer before operation.

Total patient number	76
Age (median) (IQR)	69 (64–75)
iPSA (median) (ng/mL) (IQR)	20.5 (10.8–31.6)
BMI	24.5 (22.6–27.4)
Hospital days (median) (IQR)	7 (7–9)
D’Amico risk classification	
Low	2 (3%)
Intermediate	19 (26%)
High	43 (54%)
Very high	12 (17%)
Comorbidity	
Diabetes mellitus	34 (45%)
Hypertension	16 (21%)
Cardiovascular disease	7 (9%)
Cerebrovascular accident	3 (4%)

IQR: interquartile range.

**Table 2:** Perioperative outcomes for patients with locally advanced prostate cancer.

Operative time (min) (IQR)	205 (183–241)
Mean blood loss mL (range)	66.84 ± 56.20 (30–400)
NVB sparing (%)	28 (36.8%)
LN positive (%)	37 (48.7%)
LN removal (median) (IQR)	24 (18–30) (31.6%)
Complications (%)	3 (3.9%)
Stage (%)	
IIIb	34 (44.7%)
IIIc	4 (5.3%)
IVa	38 (50.0%)
Grade group (average)	3.5
Margin-positive rate	47 (61.8%)
BCR free rate (following-up median: 36 months)	62 (72.1%)
OS rate (following-up median: 36 months)	74 (97.4%)
1-year continence rate	66 (86.8%)
3-year continence rate	69 (90.8%)
Radiotherapy	4 (5.3%)
Hormonal therapy	12 (15.8%)

IQR: interquartile range; BCR: biochemical recurrence; OS: overall survival.

## Data Availability

All of the data are available upon request to the corresponding author.
